# Peroxynitrite Is a Key Mediator of the Cardioprotection Afforded by Ischemic Postconditioning In Vivo

**DOI:** 10.1371/journal.pone.0070331

**Published:** 2013-07-10

**Authors:** Jianhui Li, Noureddine Loukili, Nathalie Rosenblatt-Velin, Pal Pacher, François Feihl, Bernard Waeber, Lucas Liaudet

**Affiliations:** 1 Department of Hepatobiliary Surgery, the First Affiliated Hospital, Zhejiang University, College of Medicine, Hangzhou, China; 2 Department of Intensive Care Medicine and Burn Center, Lausanne University Hospital Medical Center, Lausanne, Switzerland; 3 Division of Clinical Pathophysiology, Department of Internal Medicine, Lausanne University Hospital Medical Center, Lausanne, Switzerland; 4 Laboratory of Physiologic Studies, National Institute on Alcohol Abuse and Alcoholism, National Institutes of Health, Bethesda, Maryland, United States of America

## Abstract

Myocardial ischemic postconditioning (PosC) describes an acquired resistance to lethal ischemia-reperfusion (I/R) injury afforded by brief episodes of I/R applied immediately after the ischemic insult. Cardioprotection is conveyed by parallel signaling pathways converging to prevent mitochondria permeability transition. Recent observations indicated that PostC is associated with free radicals generation, including nitric oxide (NO^.^) and superoxide (O_2_^.-^), and that cardioprotection is abrogated by antioxidants. Since NO. And O_2_^. -^ react to form peroxynitrite, we hypothesized that postC might trigger the formation of peroxyntrite to promote cardioprotection in vivo. Rats were exposed to 45 min of myocardial ischemia followed by 3h reperfusion. PostC (3 cycles of 30 seconds ischemia/30 seconds reperfusion) was applied at the end of index ischemia. In a subgroup of rats, the peroxynitrite decomposition catalyst 5,10,15,20-tetrakis(4-sulphonatophenyl) porphyrinato iron (FeTPPS) was given intravenously (10 mg/kg^-1^) 5 minutes before PostC. Myocardial nitrotyrosine was determined as an index of peroxynitrite formation. Infarct size (colorimetric technique and plasma creatine kinase-CK-levels) and left ventricle (LV) function (micro-tip pressure transducer), were determined. A significant generation of 3-nitrotyrosine was detected just after the PostC manoeuvre. PostC resulted in a marked reduction of infarct size, CK release and LV systolic dysfunction. Treatment with FeTPPS before PostC abrogated the beneficial effects of PostC on myocardial infarct size and LV function. Thus, peroxynitrite formed in the myocardium during PostC induces cardioprotective mechanisms improving both structural and functional integrity of the left ventricle exposed to ischemia and reperfusion in vivo.

## Introduction

Acute myocardial infarction due to coronary artery occlusion is the major cause of chronic heart failure in developed countries. Although timely reperfusion is mandatory to rescue the ischemic tissue, it may result by itself in additional myocardial injury and dysfunction, a concept termed reperfusion injury [1]. The most powerful strategies known so far to limit ischemia-reperfusion injury involve various techniques of myocardial conditioning [2], which describes an acquired resistance to lethal ischemia/reperfusion provided by brief episodes of ischemia applied either before ischemia (preconditioning, IPC) or just before reperfusion (postconditioning, PostC) [2,3]. On a clinical viewpoint, the concept of PostC is particularly attractive, as it might be applied as a simple measure at the onset of therapeutic reperfusion, as reviewed recently [4,5]. The mechanisms underlying cardioprotection by PostC are only partially elucidated, and there seems to be many similarities with those involved in IPC [5]. The PostC manoeuvre induces the release of various autacoids, such as bradykinin, adenosine and opioids, triggering receptor-dependent activation of several parallel intracellular signalling cascades. These include the Reperfusion Injury Salvage Kinase (RISK) pathway, the Survival Activating Factor Enhancement (SAFE) pathway, protein kinase G and protein kinase C, which ultimately confer cadioprotection by inhibiting the opening of the mitochondrial permeablity transition pore thereby preventing subsequent cell death [6–8].

Recent evidence has been obtained that redox-dependent mechanisms play an important contribution to the activation of these cardioprotective signals. Both IPC and PostC are indeed associated with the generation of free radicals, including nitric oxide (NO^.^) and the superoxide radical (O_2_^.-^), and treatment with antioxidant compounds prior to the conditioning stimulus abrogate cardioprotection [9,10]. Importantly, NO and O_2_^. -^ spontaneously react, in a diffusion-controlled process, to yield the potent oxidant and nitrating species peroxynitrite [11], whose formation during IPC has been shown to be involved in its protective actions [12–14]. With respect to PostC, one study reported on an increased formation of peroxynitrite during a PostC procedure performed in an *ex vivo* rat heart preparation [15], but the potential role of peroxynitrite during *in vivo* PostC is presently unknown. Such information is critical, as it would indicate that peroxynitrite may act as a protective mediator under certain conditions, contrasting with its established cytotoxic role in the reperfused myocardium [14,16]. We therefore conducted the present study in an in vivo model of myocardial ischemia and reperfusion, in order to test the hypotheses that PostC fosters the generation of peroxynitrite in the heart to promote cardioprotection in this setting.

## Methods

### Ethic statement

The investigation conformed to the Guide for the Care and Use of Laboratory Animals published by U.S. National Institutes of Health. All animal work was conducted according to relevant national and international guidelines and was performed with the approval of the Local Institutional Animal Care and Use Committee (Service of Veterinary Affairs, State of Vaud, Switzerland, authorization Nr 1502.2).

### Animal experimental procedures

Male Wistar rats (10 weeks old, weight 250–300 g, total number = 70) were used in this study. Anesthesia was induced by intraperitoneal pentobarbital (60 mg kg^-1^.) and was maintained by subsequent doses of intraperitoneal pentobarbital (10 mg kg^-1^) according to monitoring of the depth of the anesthesia using the plantar reflex response. Anesthetized animals were then intubated, and mechanically ventilated (FiO_2_ 0.3; 65 strokes min^-1^, 8 mL kg^-1^) with a Harvard 683 rodent respirator (Holliston, MA, USA). A polyethylene (PE50) catheter was inserted into the right jugular vein for drug administration (see below). In a subset of animals, a Millar micro-tip pressure transducer was inserted into the right carotid artery for hemodynamic studies, as detailed below. Core temperature was maintained at 37±0.5°C with a heating pad. At the end of the experimental procedures, animals were euthanized with an intravenous overdose of pentobarbital (100 mg kg^-1^).

### Myocardial ischemia reperfusion

Myocardial ischemia-reperfusion (MIR) was performed according to our previously published procedure [16,17]. Briefly, the heart was exposed via a left thoracotomy and the left anterior descending coronary artery (LAD) was occluded by a small piece of PE tubing applied against the LAD by a 6-0 silk suture passed underneath the artery. After 45 minutes ischemia, reperfusion was allowed for 3 hours by relieving the PE tubing. For sham experiments, the animals underwent the same procedures with the exception that the LAD was not occluded.

## Measurements

### Left ventricular hemodynamics

Hemodynamic studies were performed in n=5 rats per group, using a micro-tip pressure catheter (SPR-671, 1.4 Fr; Millar Instruments Inc., Houston, TX), inserted into the left ventricle (LV) via the right carotid artery. Heart rate, LV end-diastolic (LVEDP) and end-systolic (LVESP) pressures were recorded, and the maximal and minimal rates of change of LV pressure (dP/dt max and dP/dt min) were calculated as load-dependent indices of LV contractility and relaxation, using a PowerLab/4SP AD converter (A D Instruments, Oxfordshire, UK).

### Determination of Myocardial Infarct Size and plasma creatine kinase activity

Area at risk (AAR) and infarct size were determined in n=7 rats/group (except from the sham group, without infarction), using the triphenyl tetrazolium chloride (TTC)-Evans blue technique, as previously described [16–18]. At the end of the experiments, heparinized whole blood was drawn from the inferior vena cava, centrifuged at 3000 rpm, and plasma was stored at -80°C. Plasma creatine kinase activity (CK) and its myocardial MB fraction (CKMB) were measured as an indicator of myocardial necrosis, using a commercial kit (Sigma Chemicals, St Louis, MO, USA).

### SDS-PAGE and western immunoblot detection of 3-nitrotyrosine in the myocardium

Groups of n=5 animals were killed after 45 minutes ischemia (or sham ischemia), just before reperfusion or immediately following PostC, and the activity of 3-nitrotyrosine (3-NT) was determined in myocardial samples from the ischemic LV as a marker of peroxynitrite generation, according to our previously published procedure [16]. Briefly, myocardial tissue was homogeneized (TrisHCl 10 mM, NP40 0.5%, NaCl 0.15 M, Na _3_VO_4_ 1 mM, NaF 10 mM, PMSF 1 mM, EDTA 1 mM, aprotinin 10 µg/ml, leupeptin 10 µg/ml, and pepstatin 1 µg/ml), and proteins (30 µg) were separated by SDS-PAGE, transferred to nitrocellulose membrane, and blocked for 1 h at room temperature with 5% nonfat dry milk in Tris-buffered saline with 0.1% Tween 20. The membrane was incubated overnight at 4°C with a 1:1000 dilution of a mouse monoclonal anti-nitrotyrosine antibody (Upstate Biotechnology, Lake Placid, NY), followed by incubation with a horseradish peroxidase–conjugated secondary antibody at a 1:5,000 dilution for 1h. The immunoblot signal was visualized using enhanced chemiluminescence (ECL, Amersham Biosciences, Otelfingen, Switzerland) and quantified by densitometric analysis. The levels of tubulin were determined in parallel as a loading control.

**Figure 1:**
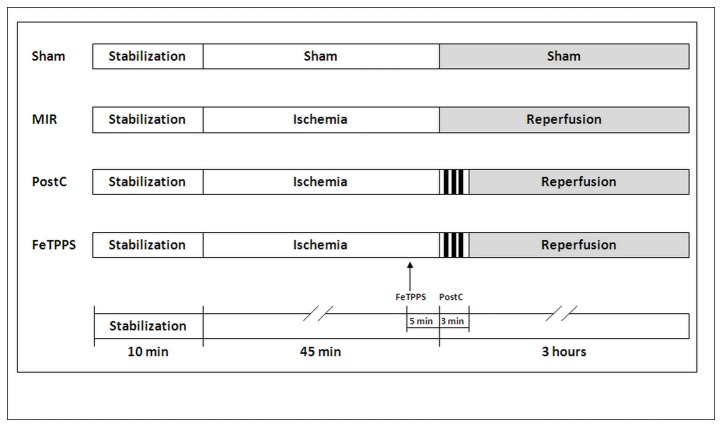
Experimental protocol (Figure 1) Four groups of rats were investigated, including Sham group, MIR group (45 minutes ischemia and 3h reperfusion), PostC group (3 cycles of 30 seconds ischemia/reperfusion applied at the end of the 45 minutes ischemia, and preceding the 3h reperfusion), and FeTPPS group (treatment with the peroxynitrite decomposition catalyst 5,10,15,20-tetrakis(4-sulphonatophenyl) porphyrinato iron (FeTPPS), 10 mg/kg intravenously 5 minutes before PostC [18]).

## Statistical analysis

All values are expressed as mean ± SD. Data were analyzed by one-way analysis of variance followed by the Dunnett’s test for multiple comparisons. A p<0.05 was considered to be statistically significant.

## Results

### Ischemic postconditioning induces 3-NT formation in the myocardium

**Figure 2:**
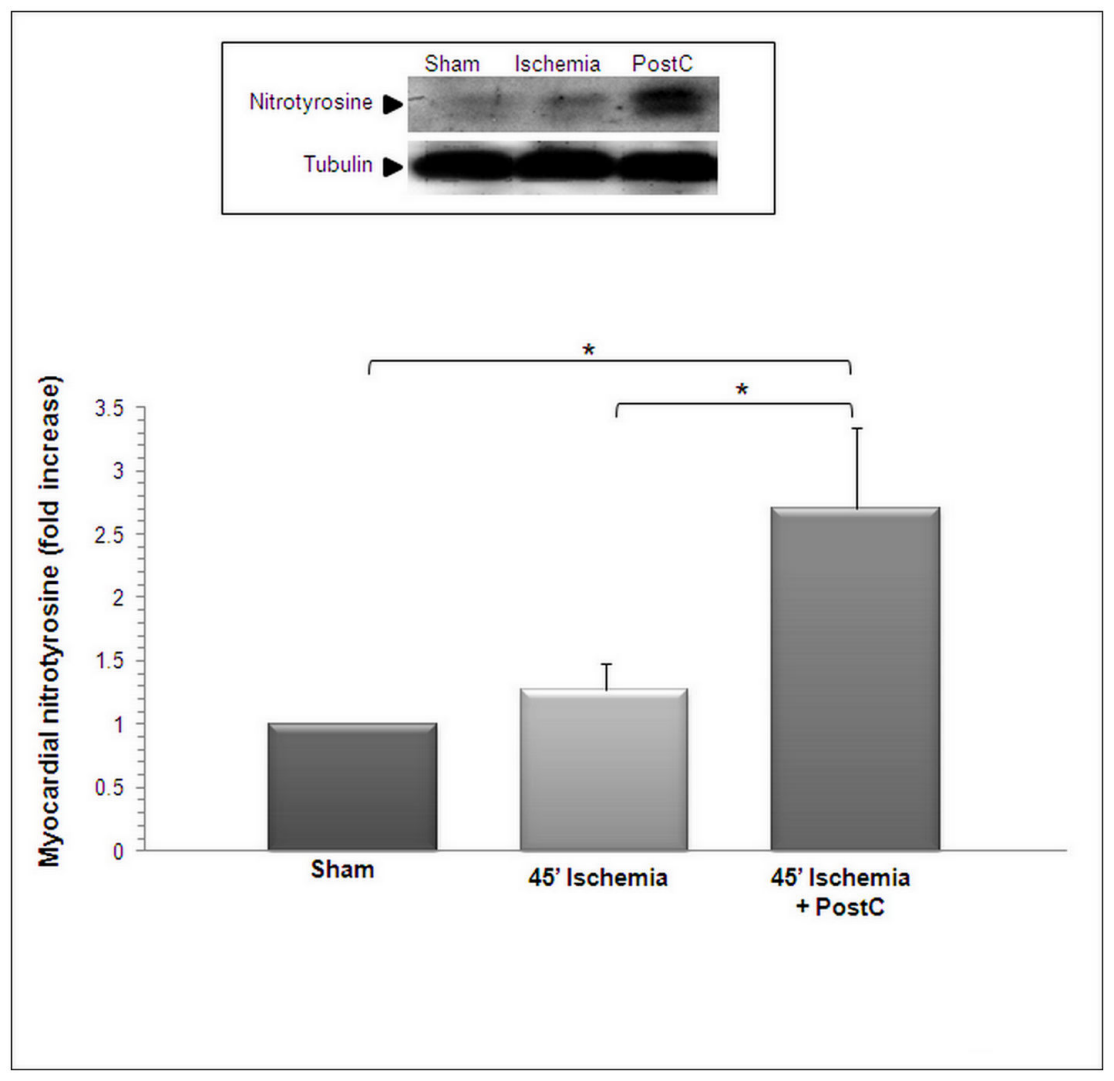
Postconditioning triggers 3-nitrotyrosine formation in the left ventricle. Nitrotyrosine was determined in the ischemic left ventricle obtained after 45 minutes sham ischemia, 45 minutes ischemia, or 45 minutes ischemia + PostC with 3 cycles of 30 seconds ischemia/reperfusion. Means ± SD of n=5 rats/condition. * p < 0.05.

As illustrated in Figure 2, there was no increased signal for 3-NT following 45 minutes of ischemia. In contrast, a marked increase of the 3-NT was detected immediately after the end of the 3 cycles of PostC, indicating that the postC manoeuvre promoted the formation of significant amounts of peroxynitrite within the ischemic LV.

### The reduction of myocardial infarct size by PostC is blunted by FeTPPS

**Figure 3:**
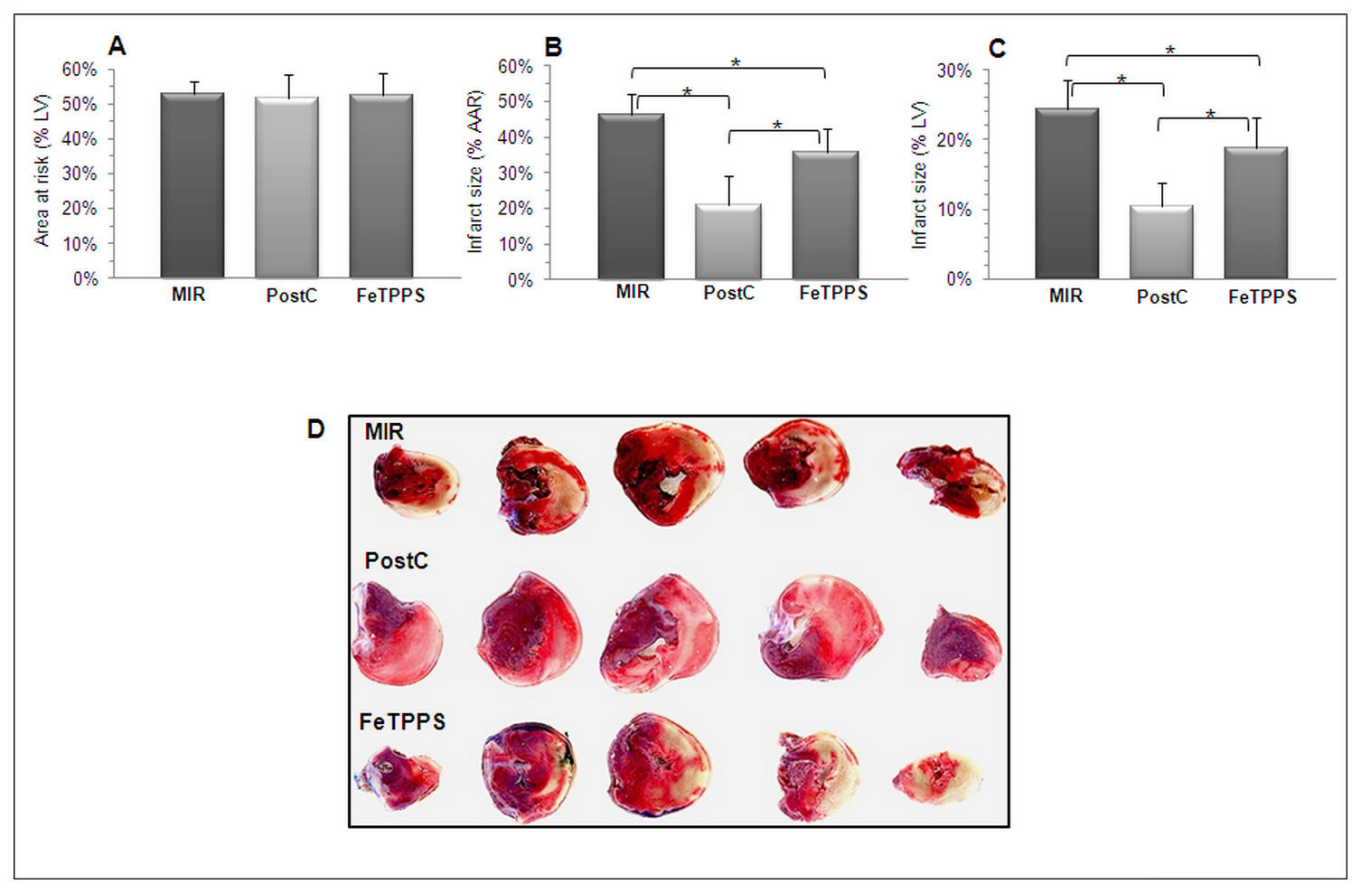
The reduction of infarct size by PostC is attenuated by FeTPPS. Rats exposed to myocardial ischemia (45 min) and reperfusion (3h) were left untreated (MIR group) or were exposed to PostC, in the absence (PostC group) or in the presence of a 10 mg/kg treatment with FeTPPS just before PostC (FeTPPS group). Area at risk (A) was comparable among the 3 groups of rats. Infarct size (B: % AAR; C: % left ventricle) was reduced by PostC, an effect suppressed by FeTPPS. D: Representative pictures of Evans blue-TTC staining of left ventricles from the the three groups of rats. Means ± SD of n=7 rats/group. * p < 0.05.

**Figure 4:**
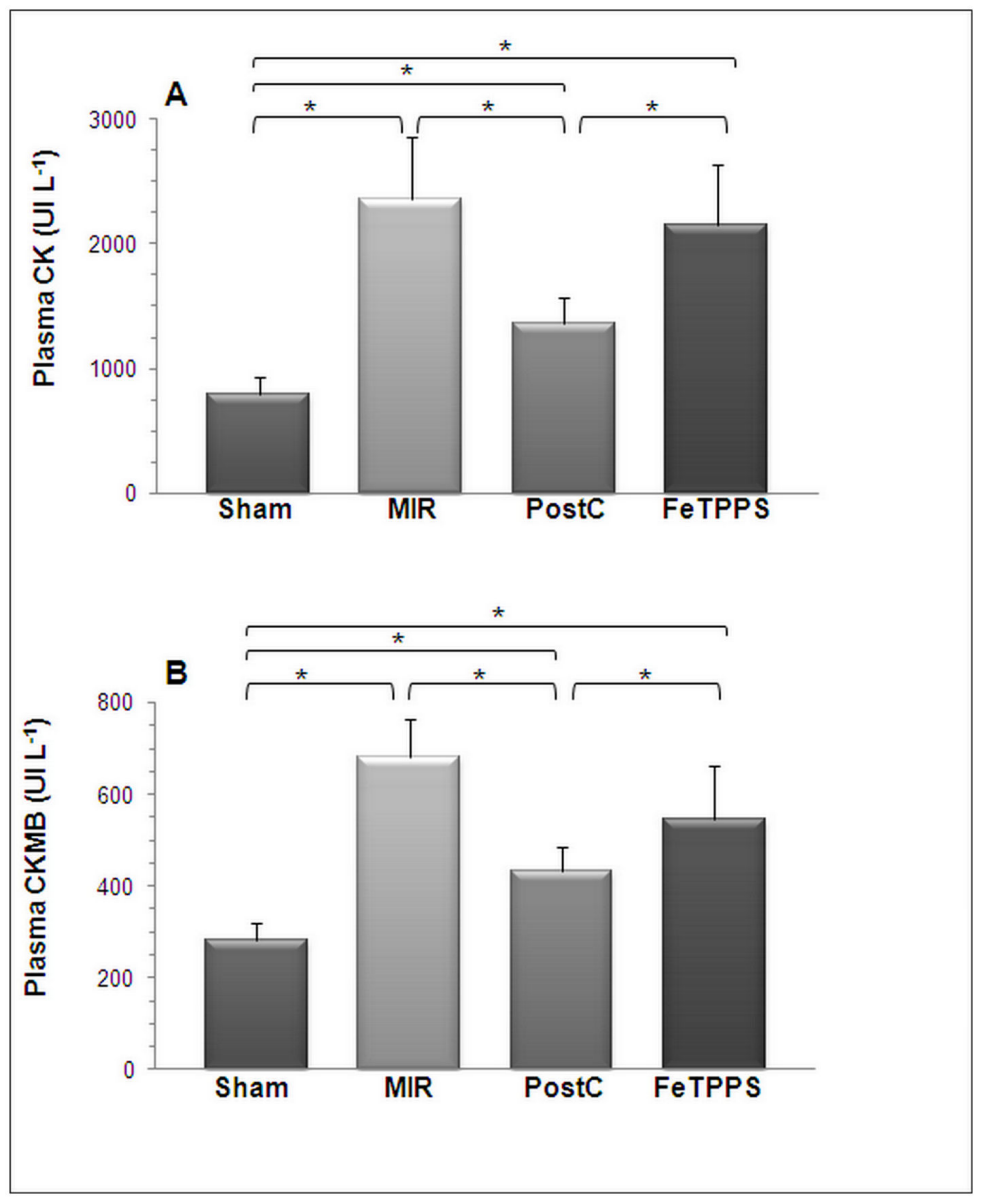
FeTPPS suppresses the effects of PostC on plasma CK and CKMB activity. Myocardial ischemia-reperfusion (MIR group) induced a large increase of plasma CK (A) and CKMB (B) activities. These increases were largely reduced by postconditioning (PostC group), but this effect was eliminated by FeTPPS treatment before PostC (FeTPPS group). Means ± SD of n=7-9 rats/group. * p < 0.05.

The area at risk (AAR, the ischemic area, Figure 3A) was comparable among the three groups of animals. Infarct size, whether expressed as a percentage of the AAR (Figure 3B), or as a percentage of the total LV (Figure 3C), was markedly and significantly reduced by PostC, an effect significantly attenuated by FeTPPS. Figure 3D shows representative images of Evans blue-TTC staining of LV from the the three groups of rats. Furthermore, the large increase of plasma CK (Figure 4A) and CKMB (Figure 4B) following MIR was significantly reduced by PostC, but this effect was abolished by FeTPPS.

### FeTPPS abolishes the benefit of PostC on post-ischemic myocardial systolic dysfunction, but not diastolic dysfunction

**Figure 5:**
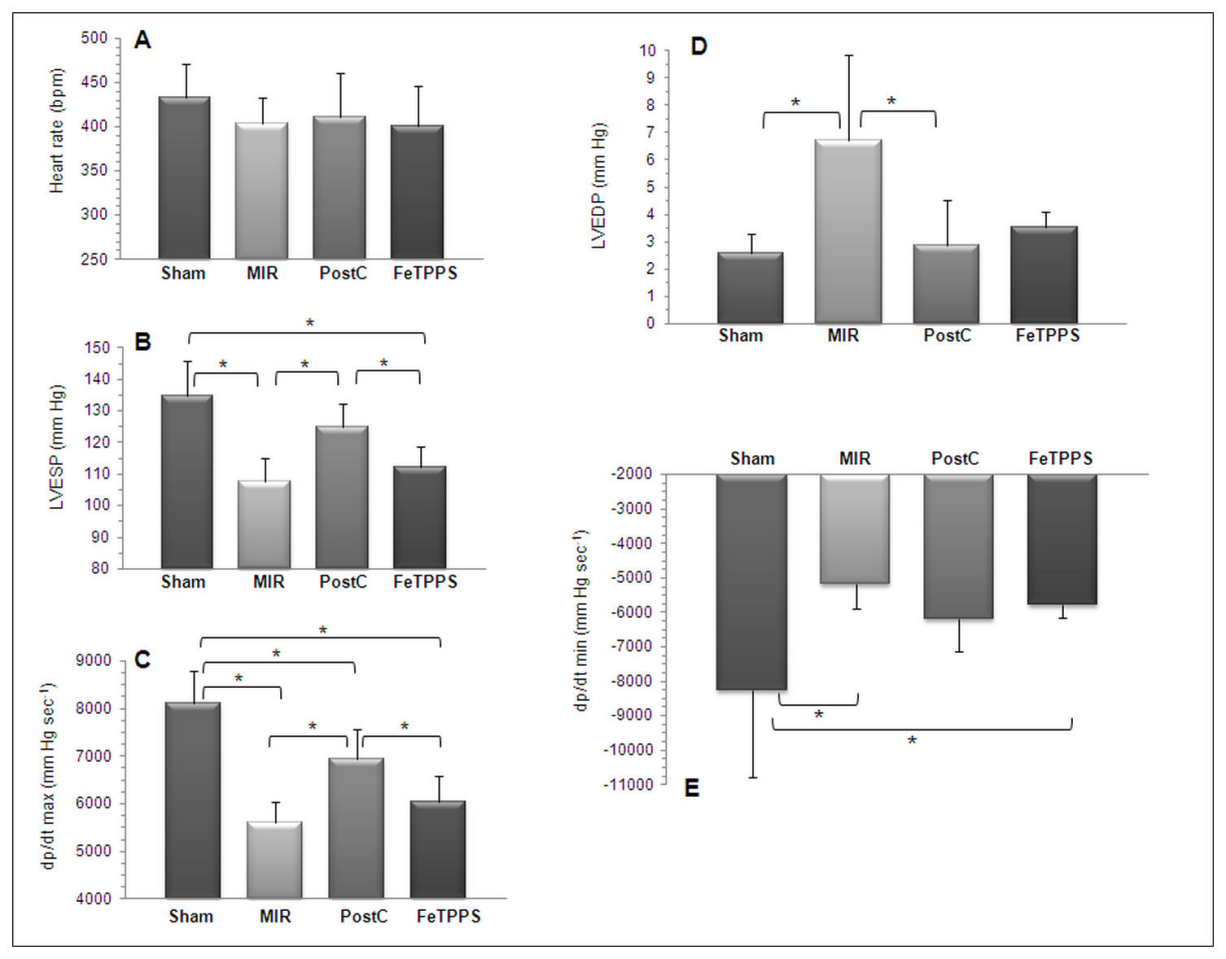
FeTPPS suppresses the beneficial effects of PostC on left ventricular contractility, but does not influence diastolic function. At the end of the observation period, heart rate was comparable among the 4 groups of rats (A). Myocardial ischemia and reperfusion (MIR group) induced a decrease of left ventricular end-systolic pressure (LVESP, B) and dp/dt max (C), which was attenuated by postconditioning (PostC group). FeTPPS suppressed the effect of PostC (FeTTPS group). Myocardial ischemia and reperfusion also induced a significant increase of left ventricular end-diastolic pressure (D: LVEDP) and a significant decrease of dp/dt min (E). PostC suppressed the increase of LVEDP, and tended to limit the reduction of dp/dt min, and these effects were not significantly influenced by FeTPPS. Means ± SD of n=5 rats/group. * p < 0.05.

As shown in Figure 5A, no significant alteration of heart rate was noted across the different experimental groups. When compared to sham animals, rats exposed to MIR disclosed a significant reduction of LVESP together with a significant drop of dp/dt max (Figure 5B and 5C), pointing to a marked decrease of LV contractility. The reduced contractility was significantly less pronounced following PostC, but this benefit was lost upon treatment with FeTPPS. Furthermore, ischemia-reperfusion also resulted in a significant impairment of diastolic relaxation, as indicated by an increased LVEDP and a reduced dp/dt min (Figure 5D and 5E). PostC suppressed the increase of LVEDP and tended to attenuate the decrease of dp/dt min, albeit nonsignificantly (p= 0.08, t test). These effects were not significantly influenced by FeTPPS.

## Discussion

The present investigation brings two important novel conclusions with respect to the mechanism of cardioprotection afforded by postconditioning *in vivo*. First, we report that a brief protocol of postconditioning is sufficient to foster the generation of significant amounts of peroxynitrite in the myocardium. Secondly, we show that the beneficial effects of postconditioning, both in terms of myocardial infarct size and cardiac systolic function, disappear when a peroxynitrite decomposition catalyst is given prior to postconditioning. Thus, our data demonstrate that peroxynitrite formation during postconditioning is a key mechanism underlying its cardioprotective actions *in vivo*.

Our protocol of postconditioning, established according to Zhao et al [3], allowed a significant reduction of myocardial infarct size, in agreement with previous investigators in a similar rat model [19]. Another important finding was the marked improvement of left ventricular systolic function, as shown by better preservation of LV systolic pressure and dp/dt max. Furthermore, PostC also improved diastolic function, indicated by reduced LV end-diastolic pressure and a blunted reduction of dp/dt min, although the latter effect was only marginally significant (p=0.08). While such functional improvement from postC has been previously reported in *ex vivo* models of myocardial ischemia [20] [21], our results indicate that PostC also ameliorates systolic and diastolic dysfunction in a relevant *in vivo* model of myocardial infarction.

Multiple identified mechanisms convey the cardioprotection of postC. Physiological mechanisms include maintenance of tissue acidosis and improved coronary endothelial function [22]. Molecular mechanisms rely on the activation of several parallel signalling pathways -protein kinase G, the RISK and SAFE pathways, protein kinase C-, conferring cardioprotection through the inhibition of mitochondrial permeability transition pore (MPTP) opening, and the activation of mitochondrial KATP channel [7,9,23]. Limited evidence has also emerged that redox-based mechanisms might be involved in posconditioning [8]. Penna et al. showed that the antioxidant N-acetylcysteine suppressed the benefit of PostC in an *ex vivo* rat model [24], whereas Tsutsumi et al. reported that the ROS scavenger 2-mercaptopropionyl glycine (MPG) suppressed the effects of PostC in an *in vivo* mouse model [25]. Furthermore, Lemoine et al. also showed that MPG abolished the effects of PostC on functional recovery of human cardiac muscle after hypoxia and reoxygenation *in vitro* [26]. Overall, these findings support the concept that the generation of oxidants during postC can represent an essential trigger of its cardioprotective effects. It has been proposed that such effects might depend on redox-mediated activation of protein kinase C and subsequent stimulation of adenosine-dependent signaling [6].

Our present findings provide additional support for a role of redox-based mechanisms in postC. First, we found that the postC manoeuvre elicited a significant generation of peroxynitrite in the myocardium, as evidenced by increased tyrosine nitration, and secondly, we found that treatment with FeTPPS just before PostC abolished the beneficial effects of the latter on infarct size and LV systolic dysfunction. Interestingly, FeTPPS did not alter the effect of PostC on diastolic dysfunction, suggesting that postC improves cardiac contractility and relaxation via distinct mechanisms, a hypothesis that should be explored in future investigations. With respect to tyrosine nitration, it is noteworthy that this post-translational modification may occur independently from peroxynitrite, via myeloperoxydase-dependent catalysis in the presence of nitrite [11]. This mechanism is however highly unlikely in the conditions of our study, since the postC manoeuvre was too brief to elicit significant leukocyte accumulation within the myocardium. We speculate that the formation of peroxynitrite during PostC was promoted owing to the simultaneous generation of its 2 precursors in the post-ischemic and immediate reperfusion phase [14]. The primary source of O_2_^. -^ in this setting is the mitochondrion, which produces a burst of O_2_^. -^ upon reoxygenation via the autooxidation of unstable semiquinones in the respiratory chain [27,28]. Regarding NO, its formation increases during ischemia, both through NOS-dependent synthesis and through the non enzymatic reduction of tissue nitrite in acidic pH [29].

Our results confirm and extend those of a previous study in an *ex vivo* model, showing that peroxynitrite played a role in the cardioprotection of PostC in isolated hearts obtained from normal, but not hyperlipidemic rats [15]. These findings may appear counterintuitive at first glance, in view of the numerous detrimental actions of peroxynitrite reported in the setting of myocardial ischemia-reperfusion injury and other heart diseases, and which include lipid peroxidation, DNA oxidative damage and activation of poly(ADP-ribose) polymerase (PARP), as well as the activation of matrix metalloproteinases, to name only a few [30,31]. However, it must be underscored that peroxynitrite does not only trigger direct cytotoxic effects, but it also promotes multiple indirect effects related to the modulation of an array of cell signaling pathways [32]. Such effects may depend either on an oxidative or a nitrative type of chemistry elicited by peroxynitrite [11], whose respective roles were not evaluated in the present study. With respect to PostC, it is here particularly noticeable that peroxynitrite has been shown to be a potent activator of ERK [33] and protein kinase C [34] in the heart, two crucial kinases involved in cardioprotection by PostC. Therefore, although our study was not designed to determine the pathways downstream of peroxynitrite-dependent protection, we may speculate on such activation of ERK and PKC as a plausible mechanism. A distinct possibility may be linked with the activation of the enzyme PARP by peroxynitrite, which has been reported to be critical for the cardioprotection elicited by preconditioning [35]. Whether a similar scenario applies to the PostC paradigm remains, however, to be established. Finally, a benefit from peroxynitrite formation during PostC might also partly depend on some preservation of the coronary endothelium and reduction of leukocyte-endothelial interactions, as supported from studies by Lefer and Nossuli et al. [36,37].

A limitation of our study is the lack of direct demonstration of peroxynitrite scavenging by FeTPPS. Although we [18,38] and others [39] previously showed that FeTPPS efficiently catalyze the decomposition of peroxynitrite, this compound may also partly react with oxidant species distinct from peroxynitrite, such as hydrogen peroxide [40]. Therefore, we cannot formally rule out that some of the observed actions of FeTPPS in the present work might have been related to effects distinct from peroxynitrite decomposition.

In conclusion, our present results indicate that peroxynitrite is a proximal mediator of cardioprotection during PostC *in vivo*. These beneficial effects contrast with the established cytotoxicity of peroxynitrite in the reperfused myocardium, which suggests that anti-oxidant strategies for therapeutic purposes might produce variable effects on the infarcted heart, depending on the time of intervention. This should be considered in future studies evaluating antioxidants for the treatment of myocardial infarction.
