# Correction: Peroxynitrite Is a Key Mediator of the Cardioprotection Afforded by Ischemic Postconditioning In Vivo

**DOI:** 10.1371/annotation/4cf3075f-3090-44d7-aa9e-8a927ed9d5fa

**Published:** 2013-07-16

**Authors:** Jianhui Li, Noureddine Loukili, Nathalie Rosenblatt-Velin, Pal Pacher, François Feihl, Bernard Waeber, Lucas Liaudet

The information for the Academic Editor of the article was omitted.

The correct information is:

Rajesh Mohanraj, UAE University, Al Ain, United Arab Emirates 

